# Association of Immunotherapy With Survival Among Patients With Brain Metastases Whose Cancer Was Managed With Definitive Surgery of the Primary Tumor

**DOI:** 10.1001/jamanetworkopen.2020.15444

**Published:** 2020-09-09

**Authors:** Saber Amin, Michael J. Baine, Jane L. Meza, Chi Lin

**Affiliations:** 1 Department of Radiation Oncology, University of Nebraska Medical Center, Omaha; 2 College of Public Health, Department of Biostatistics, University of Nebraska Medical Center, Omaha

## Abstract

**Importance:**

Immunotherapy has shown significant control of intracranial metastases in patients with melanoma. However, the association of immunotherapy combined with other cancer treatments and overall survival (OS) of patients with brain metastases, regardless of primary tumor site, is unknown.

**Objective:**

To explore the association of immunotherapy with OS in patients with cancer and brain metastases who received definitive surgery of the primary site.

**Design, Setting, and Participants:**

This comparative effectiveness study included 3112 adult patients in the National Cancer Database from 2010 to 2016 with non–small cell lung cancer, breast cancer, melanoma, colorectal cancer, or kidney cancer and brain metastases at the time of diagnosis and who received definitive surgery of the primary site. Data analysis was conducted from March to April 2020.

**Exposures:**

Treatment groups were stratified as follows: (1) any treatment with or without immunotherapy, (2) chemotherapy with or without immunotherapy, (3) radiotherapy (RT) with or without immunotherapy, and (4) chemoradiation with or without immunotherapy.

**Main Outcomes and Measures:**

The association of immunotherapy with OS was assessed with Cox proportional hazards regression, adjusted for age at diagnosis, race, sex, place of living, income, education, treatment facility type, primary tumor type, and year of diagnosis.

**Results:**

Of 3112 patients, 1436 (46.14%) were men, 2714 (87.72%) were White individuals, 257 (8.31%) were Black individuals, and 123 (3.98%) belonged to other racial and ethnic groups. The median (range) age at diagnosis was 61 (19-90) years. Overall, 183 (5.88%) received immunotherapy, 318 (10.22%) received chemotherapy alone, 788 (25.32%) received RT alone, and 1393 (44.76%) received chemoradiation alone; 22 (6.47%) received chemotherapy plus immunotherapy, 72 (8.37%) received RT plus immunotherapy, and 76 (5.17%) received chemoradiation plus immunotherapy. In the multivariable analysis, patients who received immunotherapy had significantly improved OS compared with no immunotherapy (hazard ratio, 0.62; 95% CI, 0.51-0.76; *P* < .001). Treatment with RT plus immunotherapy was associated with significantly improved OS compared with RT alone (hazard ratio, 0.59; 95% CI, 0.42-0.84; *P* = .003). Chemotherapy plus immunotherapy or chemoradiation plus immunotherapy were not associated with improved OS in the multivariable analysis.

**Conclusions and Relevance:**

In this study, the addition of immunotherapy to RT was associated with improved OS compared with radiotherapy alone in patients with brain metastases who received definitive surgery of the primary tumor site.

## Key Points

**Question:**

Is combining immunotherapy with other cancer treatments associated with improved overall survival in patients with brain metastases?

**Findings:**

In this comparative effectiveness study of 3112 adult patients who received definitive surgery of the primary cancer site, those who received any treatment plus immunotherapy had better overall survival than those who received no immunotherapy. Results varied with other combined therapies; immunotherapy plus radiation therapy was associated with improved overall survival compared with radiation therapy alone, but immunotherapy plus chemotherapy was not associated with improved overall survival compared with chemotherapy alone.

**Meaning:**

In this study, immunotherapy plus radiotherapy was associated with improved overall survival compared with radiotherapy alone.

## Teaser

This comparative effectiveness study explores the association of immunotherapy with overall survival in patients with cancer and brain metastases who received definitive surgery of the primary tumor site.

## Introduction

It is estimated that each year more than 170 000 people are newly diagnosed with brain metastases (BMs) in the United States.^1^ Brain metastases are the most common intracranial malignant neoplasms in adults and are 10-fold more common than primary intracranial cancer.^2,3,4,5^ The most common primary tumors associated with BMs are lung cancer (40%-50%), breast cancer (15%-30%), and melanoma (5%-20%), followed by colorectal cancer (3%-8%) and kidney cancer (2%-4%).^6^ Brain metastases cause significant morbidity and mortality and carry a poor survival prognosis.^7^ The median survival time is between 4 and 16 months, depending on the primary cancer site.^8,9,10^

Local therapies, such as whole-brain radiation therapy (RT), stereotactic radiosurgery, and surgical resection, have been the mainstay of treatment in these patients.^11,12^ These local therapies are associated with neurotoxic effects and represent a significant problem.^13^

Cytotoxic chemotherapy has shown limited activity in the nervous system due to its inability to cross the blood-brain barrier (BBB). With the progress in the understanding of the pathophysiology of the BBB and the development of new cytotoxic chemotherapies and targeted therapies, a renewed interest has been placed on the use of systemic treatment for BMs.^14,15^ Currently, there is a compelling rationale that drugs that sufficiently penetrate the BBB can evoke a clinical response in the central nervous system.^16^

ERBB2 inhibitors were associated with an objective central nervous system response rate of 74% and a median progression-free survival of 7.3 (95% CI, 6.5-8.1) months and a median overall survival (OS) of 10.5 (95% CI, 7.8-13.2) months in patients with breast cancer and BMs.^17^ Epidermal growth factor receptor inhibitors were associated with 3 months improved median OS in patients with BMs from non–small cell lung cancer (12 months in targeted therapy vs 9 months in chemotherapy).^18^ Targeted therapies were also associated with improved OS in patients with BMs and kidney cancer or melanoma.^19,20^ Together, these findings suggest that targeted therapies may be useful in treating intracranial disease.

Immunotherapy has changed the treatment landscape of various malignant neoplasms and may offer an exciting opportunity for the treatment of BMs.^15^ The brain was long considered as an immune-privileged organ, and it was thought that immunotherapy was ineffective in BMs because it will not cross the BBB or, when it does cross the BBB, its ability to elicit a robust immune response would be limited.^14,15^ However, preclinical and clinical evidence indicate that monoclonal antibodies can penetrate the BBB both in primary and metastatic brain cancer and thus could be an excellent option for the treatment of brain metastasis.^11,21^

**Figure 1.**
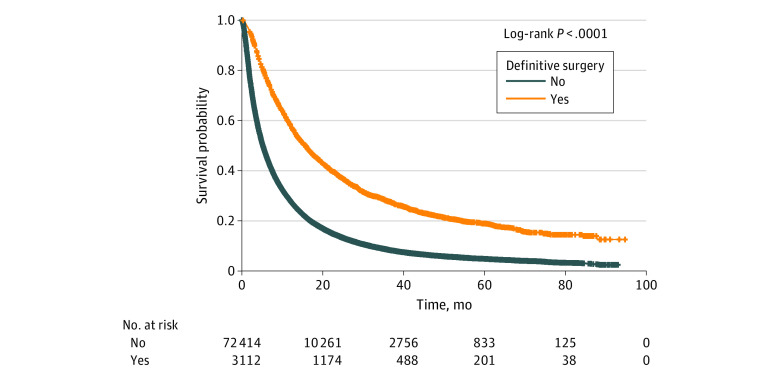
Overall Survival With or Without Definitive Surgery of the Primary Tumor Site

An ongoing phase 2 trial of pembrolizumab among patients with BMs from melanoma or non–small cell lung cancer reported a response rate of 33% (95% CI, 14%-59%) in patients with non–small cell lung cancer and 22% (95% CI, 7%-48%) in patients with melanoma.^22^ A phase 2 clinical trial of patients with BMs in which participants received a combination of nivolumab and ipilimumab reported 57% intracranial clinical benefit with 56% complete or partial responses.^23^ Retrospective studies of BMs from melanoma have also reported improved OS in patients who received immunotherapy.^24,25,26^ However, most clinical trials and retrospective studies only included BMs from melanoma, had a small number of patients, and focused on a single agent, ipilimumab.^23,24,25,26,27,28^ More extensive studies regarding the potential role of immunotherapy in patients with BMs who receive surgery of the primary tumor are lacking. The surgery distinction was made because the survival of patients with BMs who receive surgery of the primary site is different from those who do not receive surgery (20 vs 9 months) based on our data (Figure 1). Removing the primary tumor will significantly reduce the tumor burden, and immunotherapy may work better in a setting with minimal residual disease, when the bulk of the tumor mass has been optimally reduced. Using the data from the National Cancer Database (NCDB), we explored whether the use of immunotherapy in patients with BMs who received surgery of the primary site is associated with improved OS in patients with non–small cell lung cancer, breast cancer, melanoma, colorectal cancer, and kidney cancer.

## Methods

### Data Source

This was a comparative effectiveness study that used data extracted from NCDB. The NCDB is a joint program of the Commission on Cancer of the American College of Surgeons and the American Cancer Society. It captures 70% or more of newly diagnosed malignant neoplasms in the United States annually. The NCDB is a nationwide oncology outcomes database for more than 1500 Commission on Cancer–accredited cancer programs in the United States and Puerto Rico. It is the largest cancer database in the world and now contains approximately 34 million records from hospital registries from all over the United States. Participants in the NCDB database cannot be identified, neither directly nor through identifiers linked to the patients. Therefore, this research is eligible for exemption from IRB approval under 45 CFR 46.101(b)(4). The study followed the Strengthening the Reporting of Observational Studies in Epidemiology (STROBE) reporting guideline.

### Study Population

The study cohort consisted of patients with BMs aged 19 years and older who were diagnosed with the primary cancer of non–small cell lung cancer, melanoma, breast cancer, colorectal cancer, or kidney cancer between 2010 and 2016. These primary cancers account for more than 75% of BM cases. The year 2010 was chosen because this is the first year the NCDB collected information regarding BMs at the time of diagnosis. Patients who did not receive definitive surgery of the primary site and those who were missing radiation therapy, chemotherapy, and immunotherapy were excluded from the study. Patients with unknown or missing information regarding the covariates of interest were not included in the multivariable analysis. This study only includes patients with BMs who had definitive surgery of the primary cancer site. Definitive surgery refers to cancer surgery (removing primary cancer and the regional lymph nodes). Using the site-specific *International Statistical Classification of Diseases and Related Health Problems, Tenth Revision *(*ICD*-*10*) surgery codes, patients with breast cancer with codes 20 to 80, patients with non–small cell lung cancer with codes 30 to 79, patients with melanoma with codes 30 to 60, patients with colorectal cancer with codes 30 to 80, and patients with kidney cancer with codes 30 to 80 were defined as having received definitive surgery and were included in the analysis.

### End Points

The primary outcome was OS, which was calculated from the time of diagnosis of BMs to the time of death from any cause. The odds ratios for identifying the factors associated with receiving immunotherapy in the multivariable analysis were also calculated.

### Factors and Covariates

The main factors were immunotherapy, immunotherapy plus chemotherapy, immunotherapy plus RT, and immunotherapy plus chemoradiation. Covariates included the patient-level variables of age, sex, and race and the demographic variables of place of residence, income, education (based on the zip code of the patient at the time of diagnosis). The treatment-related variables of insurance, treatment facility type, comorbidity score, and receipt of immunotherapy, chemotherapy, or RT were also included.

### Statistical Analysis

The baseline covariates between patients with BMs who received immunotherapy and those who did not were compared. Mean, median, and range were calculated for all continuous variables, while proportions were reported for all categorical variables for those who received immunotherapy and for those who did not. Multiple logistic regression analysis was performed to identify the factors associated with receiving immunotherapy. The odds ratio was reported as the measure of association between the covariate of interest and the outcome of receiving immunotherapy.

The OS rates for those who received immunotherapy and those who did not receive immunotherapy were reported. The OS rates for immunotherapy plus RT vs RT alone, immunotherapy plus chemotherapy vs chemotherapy alone, and immunotherapy plus chemoradiation vs chemoradiation alone were also reported. Kaplan-Meier curves were used to report the median OS, and the log-rank test was used to indicate the significance of the findings. We also reported the Kaplan-Meier curves for the OS of patients who received immunotherapy combined with RT, chemotherapy, or chemoradiation. The Cox proportional hazard regression was used to report the hazard of death. The hazard ratio (HR) and its 95% CI for all the variables of interest were reported. Univariable Cox proportional analysis was performed for all the covariates of interest. Variables with a *P* < .15 in the univariable analysis were selected for the multivariable analysis. We performed all statistical analyses in SAS version 9.4 (SAS Institute). Statistical significance was set at *P* < .05, and all tests were 2-tailed.

## Results

### Patient and Treatment Characteristics

A total of 3112 patients diagnosed between 2010 and 2016 met the inclusion criteria and were identified from the NCDB. Of these, 1436 (46.14%) were men, 2714 (87.72%) were White individuals, 257 (8.31%) were Black individuals, 123 (3.98%) belonged to other racial and ethnic groups, 2959 (97.62%) were living in urban areas, 2924 (95.24%) had health insurance, 1196 (39.93%) were treated at academic centers, and 2254 (72.43%) had a comorbidity score of 0. The median (range) age at diagnosis was 61 (19-90) years. From 3112 patients analyzed, 183 (5.88%) received immunotherapy, 318 (10.22%) received chemotherapy alone, 788 (25.32%) received RT alone, and 1393 (44.76%) received chemoradiation alone. Overall, 22 patients (6.47%) received chemotherapy plus immunotherapy, 72 patients (8.37%) received RT plus immunotherapy, and 76 patients (5.17%) received chemoradiation plus immunotherapy.

### Outcomes

**Figure 2.**
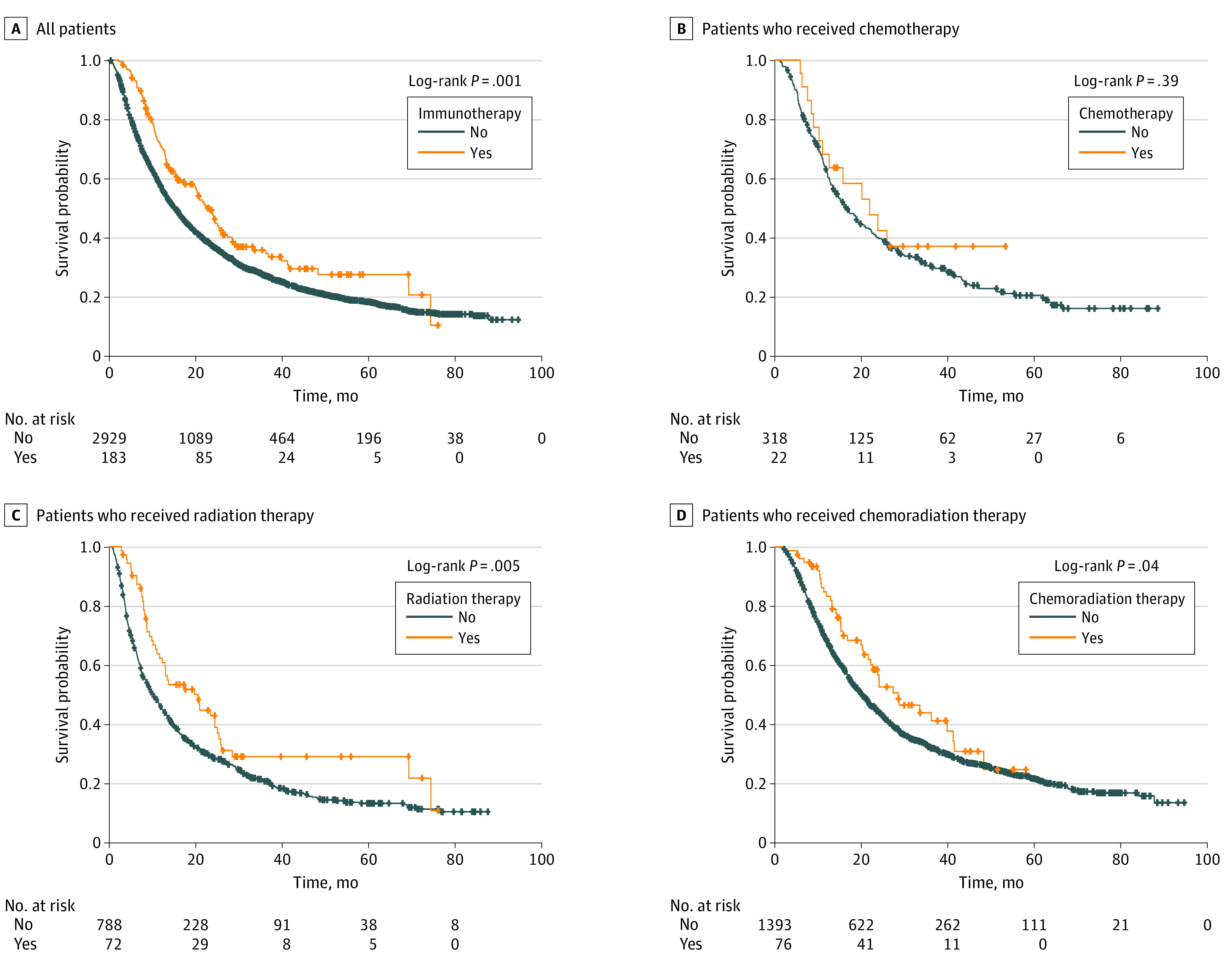
Overall Survival With or Without Immunotherapy

Patients with older age, male sex, comorbidity score of 0, diagnosis in 2014 or after, and primary cancer types of breast cancer, melanoma, and CRC (vs kidney cancer) were positively associated with the use of immunotherapy in the multivariable logistic analysis. The odds ratios of the factors associated with receiving immunotherapy are provided in Table 1. Patients who received immunotherapy had better OS compared with those who did not receive immunotherapy, with an absolute median OS benefit of 7.5 months (22.60 [95% CI, 19.71-25.92] months vs 15.08 [95% CI, 14.13-16.10] months; *P* < .001) (Figure 2A). Patients who received RT plus immunotherapy had better OS compared with patients who only received RT with an absolute median OS benefit of 10.4 months (20.53 [95% CI, 11.63-25.00] months vs 10.09 [95% CI, 8.77-11.60] months; *P* = .006) (Figure 2C). Patients who received chemoradiation plus immunotherapy had an overall benefit of 8.3 months (28.52 [95% CI, 21.59-41.23] months vs 20.21 [95% CI, 18.56-21.68] months; *P* = .04) in median OS compared with chemoradiation alone (Figure 2D). Patients who received chemotherapy plus immunotherapy did not experience survival benefit associated with the addition of immunotherapy (10.25 [95% CI, 21.91 to not reached] vs 16.66 [95% CI, 14.03-19.98]; *P* = .39) compared with chemotherapy alone (Figure 2B).

**Table 1.** Multivariable Logistic Regression Analysis of the Factors Associated With the Receipt of Immunotherapy

Variable	Patients, No. (%)			OR (95% CI)	*P* value
	Received immunotherapy (n = 183)	No immunotherapy (n = 2929)	Total (N = 3112)		
Age at diagnosis, median (range), y	58 (26-90)	61 (19-90)	61 (19-90)	0.98 (0.96-0.99)	.009
Sex					
Men	96 (52.46)	1340 (45.75)	1436 (46.14)	1 [Reference]	NA
Women	87 (47.54)	1589 (54.25)	1676 (53.86)	0.64 (0.43-0.96)	.03
Race^a^					
White	161 (87.98)	2553 (87.70)	2714 (87.72)	NA	NA
Black	13 (7.10)	244 (8.38)	257 (8.31)	NA	NA
Other	9 (4.92)	114 (3.92)	123 (3.98)	NA	NA
Unknown	0	18	18	NA	NA
County-level residents without high school diploma, %^a^					
≥13	76 (41.53)	1307 (44.70)	1383 (44.51)	NA	NA
<13	107 (58.47)	1617 (55.30)	1724 (55.49)	NA	NA
Unknown	0	5	5	NA	NA
Median county-level income, $^a^					
≥35 000	116 (63.39)	1695 (58.01)	1811 (58.33)	NA	NA
<35 000	67 (36.61)	1227 (41.99)	1294 (41.67)	NA	NA
Unknown	0	7	7	NA	NA
Place of residence^a^					
Urban	173 (96.65)	2786 (97.69)	2959 (97.62)	NA	NA
Rural	6 (3.35)	66 (2.31)	72 (2.38)	NA	NA
Unknown	4	77	81	NA	NA
Hospital type					
Academic	83 (48.82)	1113 (39.40)	1196 (39.93)	1 [Reference]	NA
Community	87 (51.18)	1712 (60.60)	1799 (60.07)	0.72 (0.52-1.01)	.06
Unknown	13	104	117	NA	NA
Insurance status^a^					
Insured	178 (97.27)	2746 (95.12)	2924 (95.24)	NA	NA
Not insured	5 (2.73)	141 (4.88)	146 (4.76)	NA	NA
Unknown	0	42	42	NA	NA
Charlson/Deyo score					
0	152 (83.06)	2102 (71.77)	2254 (72.43)	1 [Reference]	NA
≥1	31 (16.94)	827 (28.23)	858 (27.57)	0.65 (0.43-0.99)	.04
Chemotherapy^a^					
Yes	98 (53.55)	1711 (58.42)	1809 (58.13)	NA	NA
No	85 (45.45)	1218 (41.52)	1303 (41.87)	NA	NA
Radiation therapy					
Yes	148 (80.87)	2181 (74.46)	2329 (74.84)	1 [Reference]	NA
No	35 (19.13)	748 (25.54)	783 (25.16)	0.63 (0.42-0.95)	.03
Cancer type					
Breast	43 (23.50)	560 (19.12)	603 (19.38)	2.42 (1.36-4.31)	.003
NSCLC	24 (13.11)	1142 (38.99)	1166 (37.47)	0.44 (0.26-0.76)	.003
Melanoma	55 (30.05)	231 (7.89)	286 (9.19)	4.99 (3.10-8.02)	.001
CRC	24 (13.11)	326 (11.13)	350 (11.25)	1.83 (1.04-3.24)	.04
Kidney	37 (20.22)	670 (22.87)	707 (22.72)	1 [Reference]	NA
Year of diagnosis					
2010-2013	83 (45.36)	2036 (69.51)	2119 (68.09)	0.32 (0.23-0.44)	<.001
2014-2016	100 (54.64)	893 (31.49)	993 (31.91)	1 [Reference]	NA

Abbreviations: CRC, colorectal cancer; NA, not applicable; NSCLC, non–small cell lung cancer; OR, odds ratio.

^a^ The variables of race, education, income, place of residence, insurance status, and chemotherapy were not included in the multivariable logistic regression analysis above because each of them had a *P* > .15 in the univariate analysis.

In the univariable Cox proportional hazard analysis (Table 2), patients who received immunotherapy had improved OS compared with their counterparts (HR, 0.73; 95% CI, 0.60-0.88; *P* < .001). We also found significantly improved OS among patients who received RT plus immunotherapy vs RT alone (HR, 0.66; 95% CI, 0.49-0.89; *P* = .006) and chemoradiation plus immunotherapy vs chemoradiation alone (HR, 0.72; 95% CI, 0.53-0.99; *P* = .04) (Table 3). Younger age, female sex, living in areas with median income of $35 000 or greater, receiving treatment at academic centers, having a comorbidity score of 0, receiving chemotherapy, receiving RT, and having a tumor type of non–small cell lung cancer were also associated with improved OS in the univariable analysis (Table 2).

**Table 2.** Univariable and Multivariable Cox Proportional Regression Analysis of Factors Associated With Overall Survival

Variable	Univariable analysis		Multivariable analysis	
	HR (95% CI)	*P* value	HR (95% CI)	*P* value
Age at diagnosis, continuous	1.02 (1.02-1.03)	.001	1.02 (1.01-1.02)	.001
Sex				
Men	1 [Reference]	NA	1 [Reference]	NA
Women	0.88 (0.81-0.96)	.003	0.94 (0.86-1.04)	.24
Race				
White	1 [Reference]	NA	1 [Reference]	NA
Black	1.07 (0.93-1.24)	.36	1.09 (0.93-1.27)	.28
Other	0.75 (0.59-0.94)	.01	0.81 (0.64-1.04)	.09
County-level residents without high school diploma, %^a^				
≥13	1.06 (0.97-1.15)	.19	NA	NA
<13	1 [Reference]	NA	NA	NA
Median county-level income, $^a^				
≥35 000	1 [Reference]	NA	1 [Reference]	NA
<35 000	1.14 (1.05-1.23)	.003	1.07 (0.98-1.17)	.12
Place of residence^a^				
Urban	1 [Reference]	NA	NA	NA
Rural	1.10 (0.85-1.43)	.46	NA	NA
Hospital type				
Academic	1 [Reference]	NA	1 [Reference]	NA
Community	1.36 (1.25-1.49)	.001	1.27 (1.16-1.38)	.001
Insurance status^a^				
Insured	1 [Reference]	NA	NA	NA
Not insured	1.08 (0.89-1.32)	.42	NA	NA
Charlson/Deyo score				
0	1 [Reference]	NA	1 [Reference]	NA
≥1	1.23 (1.12-1.34)	.001	1.15 (1.05-1.26)	.004
Year of diagnosis^a^				
2014-2016	1 [Reference]	NA	NA	NA
2010-2013	1.03 (0.94-1.13)	.54	NA	NA
Type of cancer				
Kidney	1 [Reference]	NA	1 [Reference]	NA
Breast	0.92 (0.81-1.04)	.18	0.96 (0.83-1.12)	.60
NSCLC	0.74 (0.66-0.83)	.001	0.80 (0.71-0.90)	.001
CRC	1.33 (1.14-1.56)	.001	1.43 (1.21-1.69)	.001
Melanoma	1.86 (1.62-2.14)	.001	1.97 (1.70-2.29)	.001
Chemotherapy				
Yes	0.61 (0.56-0.66)	<.001	0.68 (0.62-0.75)	.001
No	1 [Reference]	NA	1 [Reference]	NA
Radiation therapy				
Yes	0.79 (0.72-0.87)	<.001	0.94 (0.85-1.04)	.21
No	1 [Reference]	NA	1 [Reference]	NA
Immunotherapy				
Yes	0.73 (0.60-0.88)	<.001	0.62 (0.51-0.76)	.001
No	1 [Reference]	NA	1 [Reference]	NA

Abbreviations: CRC, colorectal cancer; HR, hazard ratio; NA, not applicable; NSCLC, non–small cell lung cancer.

^a^ These variables were not included in the multivariable analysis because they had *P* > .15 in the univariable analysis.

**Table 3.** Univariate and Multivariate Analysis of Combining Immunotherapy With Chemotherapy and Radiation Therapy

Group	No. (%)	Univariable analysis		Multivariable analysis^a^	
		HR (95% CI)	*P* value	HR (95% CI)	*P* value
Chemotherapy treatment group (n = 340)					
Chemotherapy only	318 (93.53)	1 [Reference]	NA	1 [Reference]	NA
Chemotherapy with immunotherapy	22 (6.47)	0.79 (0.45-1.37)	.40	0.79 (0.44-1.42)	.43
Radiotherapy treatment group (n = 860)					
Radiotherapy only	788 (91.63)	1 [Reference]	NA	1 [Reference]	NA
Radiotherapy with immunotherapy	72 (8.37)	0.66 (0.49-0.89)	.006	0.59 (0.42-0.84)	.003
Chemoradiation treatment group (n = 1469)					
Chemoradiation only	1393 (94.83)	1 [Reference]	NA	1 [Reference]	NA
Chemoradiation with immunotherapy	76 (5.17)	0.72 (0.53-0.99)	.04	0.75 (0.54-1.03)	.07

Abbreviations: HR, hazard ratio; NA, not applicable.

^a^ Three different models were developed for the multivariable analysis because the groups were mutually exclusive. All three models were adjusted for age at diagnosis, race, hospital type, comorbidity score, and tumor type. The chemotherapy plus immunotherapy model also included income, while the chemoradiation plus immunotherapy model included income and year of diagnosis in addition to other factors.

In the multivariable analysis (Table 2) adjusted for age at diagnosis, sex, income, hospital type, comorbidity score, and receipt of chemotherapy or RT, patients who received immunotherapy had significantly improved OS compared with no immunotherapy (HR, 0.62; 95% CI, 0.51-0.76; *P* < .001). Treatment with RT plus immunotherapy was associated with significantly improved OS compared with RT alone (HR, 0.59; 95% CI, 0.42-0.84; *P* = .003) in the multivariable analysis. Chemotherapy plus immunotherapy and chemoradiation plus immunotherapy were not associated with improved OS in the multivariable analysis (Table 3). Younger age, academic hospital type, comorbidity score of 0, receiving chemotherapy, and tumor type of non–small cell lung cancer were associated with improved OS in the multivariable analysis.

## Discussion

In the current analysis, we found significant OS benefit for patients who received immunotherapy combined with RT compared with RT alone. Younger age, receiving treatment at an academic center, comorbidity score of 0, primary cancer of non–small cell lung cancer, and use of RT were associated with improved OS. Median OS was longer in patients who received RT plus immunotherapy compared with patients who only received RT alone. Most importantly, we found that immunotherapy improved the OS of patients with BMs by 7.5 months regardless of what other treatments they received. Immunotherapy combined with RT improved OS by 10 months compared with those who only received RT.

The median survival time reported in our study is comparable to the median survival time indicated in previous studies.^29,30,31^ In the multivariable analysis in this study, we found that RT plus immunotherapy was associated with significantly improved OS compared with RT alone (HR, 0.59; 95% CI, 0.42-0.84; *P* = .003). Previously published studies have also investigated the association of immunotherapy combined with RT and chemotherapy with outcomes.^17,18,19,20,21,22,23,24,25,26,27,28,29,30,31,32,33,34,35,36,37^ Most previous studies only included patients with metastatic melanoma who received a single drug (ie, ipilimumab).^28,35,36,37^ The findings of the current study are consistent with the earlier studies that were focused on patients with melanoma.^23,25,26,28,29,30,31,32,35,36,37^ However, based on our knowledge, none of the previous studies included patients with non–small cell lung cancer, breast cancer, small cell lung cancer, CRC, or kidney cancer.

What is unique in our study is that we found that immunotherapy was associated with significantly improved survival in patients with BMs who received surgery of the primary site, which has not, to our knowledge, been investigated so far. The improved OS with the addition of immunotherapy to RT may indicate the synergetic or additive effect of immunotherapy with RT. The improved OS in patients who received RT plus immunotherapy may be associated with the abscopal effect of RT. After a tumor is irradiated, injury in the tumor may lead to the release of tumor-associated antigens, which can stimulate a tumor-specific immune response, allowing the immune cells (ie, T-cells) to recognize and attack both the primary tumor and metastatic disease in a sort of autovaccination.^38,39,40,41^ Immunotherapy may enhance the optimal effect of the abscopal effect by increasing and improving the immune response to tumor-associated antigens, notably when the removal of the primary tumor minimizes the tumor burden. Radiation therapy also causes the release of neoantigens and upregulation of inflammatory cytokines, which promote the presentation of the neoantigens in the tumor microenvironment and thereby increase the immunogenicity of the tumor cells, making them a better target for immunotherapy.^42,43,44^ For patients with significant extracranial disease, the addition of immunotherapy may improve survival by controlling extracranial disease. However, for those without or with minimal extracranial disease, the association of immunotherapy with survival will be mediated through the control of BMs, which will be influenced by the drug permeability of BBB.

To our knowledge, this study is the first to use an extensive database such as NCDB and to investigate the association of immunotherapy combined with chemotherapy and/or RT with the OS of patients with BMs who received definitive surgery of the primary tumor. The findings of our study, together with the results of the previously published studies of immunotherapy in patients with melanoma, warrant future clinical trials of immunotherapy combined with chemotherapy and/or RT in patients who receive definitive surgery of the primary tumor. Patients with BMs have been historically excluded from clinical trials of immunotherapy due to poor survival and fear of adverse effects. For patients with BMs who do not participate in clinical trials, proper treatment of BM still requires multidisciplinary input regarding appropriate integration from surgery, radiation, and systemic therapies. Furthermore, the quality of life of patients and long-term toxic effects should be carefully weighed when immunotherapy is recommended. Immunotherapy is not a standard-of-care treatment in BMs outside of clinical trials. However, some patients are receiving immunotherapy. These patients may have been taking part in a clinical trial and received immunotherapy as part of a study. Patients who participate in trials are highly selected populations and usually have better prognoses. However, in our study, 83 of 170 patients (48.82%) for whom information regarding treatment facility was available were treated at academic centers, an indication that most patients may have been treated at community centers and may not have been participating in clinical trials. However, we cannot determine whether a patient was participating in a clinical trial by using the treatment facility variable. It is also possible that immunotherapy was recommended for patients who could not tolerate standard-of-care treatments or failed to show any response to standard-of-care treatments. Last but not least, immunotherapy could be recommended for patients who have exhausted many lines of standard-of-care treatments. The strength of this study is the large sample size and the inclusion of patients with various primary tumors.

### Limitations

The current study is not without limitations. The limitations are those that are inherent to any secondary analysis of an extensive database such as NCDB and include lack of data regarding the cause of death, type of immunotherapy, lack of information regarding chemotherapy regimens, incomplete data, and ascertainment bias. The immunotherapy group represented only 5.7% of patients who received definitive surgery of the primary tumor site, indicating that this is a highly select group of patients with BMs and many of these patients might have been enrolled in clinical trials. This group may also have characteristics that we were not able to adequately account for from the database, including the decision-making regarding the use of immunotherapy. Not knowing whether patients have received surgery to the brain is another limitation of the current study because the NCDB does not have information on surgery to intracranial lesions for patients with BMs. Another limitation is the lack of information regarding the numbers of intracranial lesions, the extent of the intracranial tumor, and the size of the intracranial tumor. Also, not analyzing whether RT was given intracranially, extracranially, or both and whether it was fully fractionated RT, stereotactic radiosurgery, or stereotactic body RT are other vital limitations and may cause selection bias. Although we found that immunotherapy was associated with improved OS, it is not clear whether improved OS was owing to treating extracranial disease, reducing the incidence of new brain metastasis, or both. Nevertheless, the study analyzed the data from the best available cancer database available in the United States outside the setting of multicenter clinical trials. It investigated the association of immunotherapy with the survival of patients with BMs who received definitive surgery of the primary tumor.

## Conclusions

In this study, we found that immunotherapy combined with RT was significantly associated with improved OS. The findings warrant future clinical trials investigating the association of chemotherapy, RT, and chemoradiation combined with immunotherapy with the survival of patients who receive definitive surgery of the primary tumor.
